# Identification of Key PANoptosis Regulators in Periodontitis and Chronic Obstructive Pulmonary Disease Using Gene Expression and Machine Learning Methods

**DOI:** 10.3390/genes16091027

**Published:** 2025-08-29

**Authors:** Suheyla Kaya, Nail Besli, Ilhan Onaran

**Affiliations:** 1 Department of Periodontology, Faculty of Dentistry, Istanbul University—Cerrahpaşa, Istanbul 34098, Türkiye; 2 Department of Medical Biology, Hamidiye School of Medicine, University of Health Sciences, Istanbul 34668, Türkiye; beslinail@gmail.com; 3 Medical Department of Biology, Department of Basic Medical Sciences, Faculty of Medicine, Istanbul University—Cerrahpaşa, Istanbul 34098, Türkiye

**Keywords:** chronic obstructive pulmonary disease, core genes, differentially expressed genes, immune infiltration, PANoptosis, periodontitis, machine learning

## Abstract

Background: Periodontitis (PD) is a chronic inflammatory disease associated with systemic conditions such as chronic obstructive pulmonary disease (COPD). PANoptosis—a form of regulated cell death integrating pyroptosis, apoptosis, and necroptosis—has been implicated in inflammatory diseases, but its role in PD and its overlap with COPD is not well understood. Methods: Gene expression datasets for PD and COPD were retrieved from the Gene Expression Omnibus (GEO). Differentially expressed genes were intersected with 78 PANoptosis-related genes. Functional enrichment (GO, KEGG), protein–protein interaction (PPI) network analysis, and machine learning (XGBoost with ROC curves) identified key regulatory genes. Immune infiltration was evaluated, and drug–gene interactions were analyzed using DGIDB. Results: Seven PANoptosis-related core genes—*ACO1*, *NLRC4*, *CASP8*, *HSPA4*, *IL1B*, *MEFV*, and *CYCS*—were identified in both PD and COPD. These genes were enriched in pathways involving inflammasomes, apoptosis, and oxidative stress. Immune analysis showed significant differences in B cells, T cells, dendritic cells, and plasma cells. Potential drug targets, including *IL1B* and *CASP8*, were identified. Conclusions: This is the first study to link PANoptosis to both PD and COPD. The findings reveal shared molecular mechanisms and suggest PANoptosis-related genes as novel biomarkers and therapeutic targets in chronic inflammatory oral disease.

## 1. Introduction

PANoptosis (Pyroptosis, Apoptosis, and Necroptosis), a newly identified form of programmed cell death (PCD), holds significant pathophysiological relevance to inflammation and autoimmunity. As a unique innate immune-inflammatory PCD pathway, it reveals the crosstalk and redundancy among different forms of PCD, thereby providing a deeper understanding of the link between innate immunity and cell death. PANoptosis is closely associated with infections, autoimmune diseases, and inflammation [1].

Periodontitis (PD) is an inflammatory disease of the tissues supporting the teeth, caused by a dysbiosis between oral bacteria and the host immune system, and is associated with systemic low-grade inflammation, affecting 45–50% of adults globally. Its severe form is considered the sixth most common human disease, impacting 11.2% of the adult population. Characterized by chronic inflammation, PD extends beyond the periodontal tissues, entering the vascular system and increasing systemic inflammation, which may contribute to other systemic diseases. Evidence links PD to various chronic non-communicable diseases including diabetes, cardiovascular diseases, respiratory diseases, adverse pregnancy outcomes, and Alzheimer’s disease. Recent studies suggest that the systemic low-grade inflammation caused by PD can trigger trained innate immunity, a long-lasting, heightened immune response resulting from changes in early blood-forming cells in the bone marrow. This process leads to trained myelopoiesis, where the bone marrow continuously produces more inflammatory cells, contributing to chronic inflammation. This may help explain how PD contributes to other inflammatory diseases. The relationship is bidirectional, as systemic diseases can also increase the risk of developing PD. Therefore, PD may not be just a local oral disease, but a contributor to systemic inflammation through trained innate immunity. Among the systemic effects of PD, its association with respiratory diseases has also drawn attention [2]. In this context, the most recent systematic review and meta-analysis examining the relationship between respiratory diseases and PD has highlighted a positive association between PD and chronic obstructive pulmonary disease (COPD) [3].

COPD, the third leading cause of death globally, is characterized by high morbidity and mortality, posing a major disease burden. Chronic inflammation plays a central role in the onset and progression of COPD, primarily affecting the lung parenchyma and surrounding airways. This leads to persistent respiratory symptoms and irreversible airflow limitation [4]. Activation of the innate immune system by damage-associated signals results in sustained inflammation and impaired pathogen clearance. This chronic immune activation contributes to tissue damage and airway remodeling, which are key drivers of COPD progression [5].

The link between PD and respiratory diseases, such as COPD, is thought to involve two potential mechanisms: (1) micro-aspiration of oral pathogens into the lower airways, and (2) low-grade systemic inflammation induced by PD, which, along with cytokine-mediated effects on pulmonary epithelial cells, may exacerbate respiratory conditions. Common risk factors such as smoking, obesity, and diabetes complicate the relationship and should be considered when investigating the underlying biological mechanisms. While the precise mechanisms remain unclear, further research is needed to establish causality [6]. Conversely, COPD may exacerbate PD through several interrelated mechanisms. The use of inhaled medications, such as corticosteroids and bronchodilators, can decrease salivary flow and alter the oral environment, facilitating microbial dysbiosis and increasing susceptibility to periodontal inflammation. Furthermore, systemic immune dysregulation and oxidative stress associated with COPD may impair periodontal tissue homeostasis and accelerate disease progression [7].

A novel area of research that may offer insight into this link is PAN-optosis, a form of PCD that integrates aspects of pyroptosis, apoptosis, and necroptosis into a single complex process mediated by the PANoptosome. The full range of biological effects observed in PANoptosis cannot be explained by pyroptosis, apoptosis, or necroptosis alone. It is defined as an innate immune, lytic, and inflammatory cell death pathway driven by caspases and RIPKs, and regulated by PANoptosome complexes [8]. The PANoptosome is composed of sensors, adapters, and effectors that coordinate these three forms of PCD, which were previously thought to be independent [9]. In response to microbial infections or disrupted cellular homeostasis, sensors such as ZBP1, AIM2, RIPK1, and NLRP12 interact to assemble PANoptosomes, which recruit key molecules including caspases, RIPKs, and ASC. These include ZBP1-PANoptosome, AIM2-PANoptosome, RIPK1-PANoptosome, and NLRP12-PANoptosome. The formation of these complexes activates caspase-3/7, cleaves GSDMD/GSDME, and phosphorylates MLKL, leading to membrane pore formation and PANoptosis progression [8].

The intricate interplay of PCD pathways in PD remains largely unexplored. Although previous studies have examined individual PCD modalities—such as apoptosis, pyroptosis, or necroptosis—mounting evidence suggests that these pathways are not isolated but instead engage in extensive crosstalk, forming a coordinated system with the capacity for mutual compensation. However, the full range of biological effects observed in inflammatory responses cannot be adequately explained by any single PCD pathway [10]. Since the mechanisms of PD cannot be fully explained by a single form of PCD, we aimed to investigate it through the lens of PANoptosis. A comprehensive understanding of the roles of PCD in microbial balance and periodontal tissue homeostasis may offer novel therapeutic strategies for PD. Unlike previous bioinformatic studies that focused on isolated PCD types, our study highlights the regulators of PANoptosis, a novel and comprehensive approach that provides deeper insights into the immunopathogenesis of PD and identifies potential targets for therapeutic intervention.

Although there are no studies yet that exclusively examine PANoptosis in the context of PD, existing research indicates that the components of the PANoptosome are highly expressed in periodontal tissues, suggesting that this process may contribute to periodontal inflammation and tissue destruction [11]. In parallel, COPD has also been shown to be closely associated with PANoptosis, which plays a pivotal role in the progression of pulmonary inflammation and damage. Given the shared inflammatory mechanisms between PD and COPD, including the potential involvement of PANoptosis, further exploration of these pathways could provide new insights into the pathophysiology of both diseases [12].

To explore these mechanisms, we employed bioinformatics approaches to integrate clinical data from PD and COPD. By identifying cross-talk genes between the two diseases and linking them to PANoptosis-related genes through correlation analysis and protein–protein interaction (PPI) networks, we hypothesize key genes involved in the relationship between these disorders. Additionally, this study investigates immune cell infiltration and assesses the potential of these genes as diagnostic biomarkers and therapeutic targets. While conventional periodontal treatments primarily focus on mechanical removal of bacterial deposits, targeting the inflammatory response, especially those involving PANoptosis, may provide a more holistic approach to managing PD and its systemic implications, including its relationship with COPD. Identifying shared genetic markers and inflammatory pathways between PD and COPD could inform strategies for controlling both local and systemic inflammation, ultimately improving patient outcomes.

## 2. Materials and Methods

### 2.1. Data Accession and Preprocessing

Gene expression data for PD and COPD were retrieved from the Gene Expression Omnibus (GEO) database [13]. For microarray datasets, log2 transformation was applied following standard preprocessing. This normalization was performed prior to batch correction and log2 transformation to stabilize variance. The final merged matrix was then corrected for batch effects using the COMBAT method from the sva package. The datasets were identified using the keywords “Periodontitis” for PD and “Chronic Obstructive Pulmonary Disease” for COPD. Inclusion criteria required datasets to originate from Homo sapiens and involve experimental methods such as microarrays. Following these criteria, two PD-related datasets (GSE16134, and GSE10334) were selected. Similarly, four COPD-related datasets (GSE27597, GSE38974, GSE76925, and GSE106986) were acquired. All datasets pertained to gene expression data from disease groups (PD and COPD) and their corresponding healthy controls. These datasets were used to analyze differentially expressed genes (DEGs).

**Figure 1:**
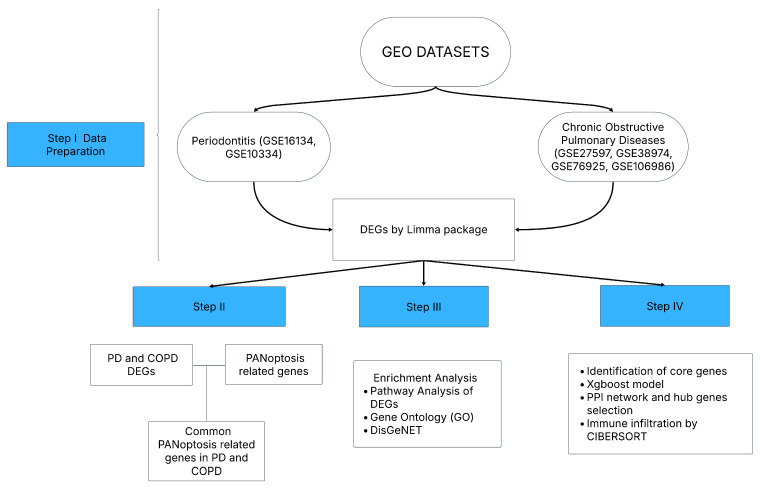
Flowchart of the study.

The study’s workflow is outlined in Figure 1, and the detailed characteristics of the included datasets are summarized in Table 1. Using the data from these datasets, intersecting genes across each disease condition were identified. The PD and COPD datasets were combined separately, and batch effects were corrected using the COMBAT method from the sva package (version 3.54.0) in RStudio (version 4.4.1). The batch effect correction aimed to minimize technical variation between datasets and create a more consistent dataset for downstream analyses. All downstream analyses, including DEG identification, Receiver Operating Characteristic (ROC) analysis, immune infiltration, and classification, were performed on these merged and batch-corrected disease-specific expression matrices.

### 2.2. Acquiring Genes Associated with PANoptosis

To identify common genes related to PANoptosis between PD and COPD and perform a correlative analysis, we conducted DEG analysis separately on each dataset using the GEO2R tool (https://www.ncbi.nlm.nih.gov/geo/geo2r, accessed on 30 October 2024). DEG analysis was performed to identify genes that are differentially expressed in PD and COPD compared to healthy controls. PANoptosis-related genes were identified within these datasets by cross-referencing the DEG results with gene lists associated with PANoptosis from previous studies (PMID: 37895022 and PMID: 37077221). These genes were further validated by checking their interactions in the Search Tool for the Retrieval of Interacting Genes/Proteins (STRING) [14] database, which provides functional protein association networks. Genes that intersected with those listed in the STRING database under the specified PMIDs were designated as common genes associated with the PANoptosis cellular process.

### 2.3. Differentially Expressed Genes Analysis

After batch correction, differential expression analysis was performed on the merged microarray datasets using the limma package (version 3.62.1). For both the PD and COPD groups, differentially expressed genes (DEGs) were identified by comparing case and control samples, applying a threshold of *p*-value < 0.05 and |log fold change (FC)| > 0.5. Volcano plots were used to visualize DEG distributions, generated using the ggpubr (version 0.6.0) and ggthemes (version 5.1.0) packages. This approach ensured robust statistical modeling and consistent analysis across all included datasets.

### 2.4. Functional Enrichment Analysis of Differentially Expressed Genes

In this study, we utilized the Metascape database [15] to perform functional enrichment analyses of the DEGs associated with PD and COPD. Metascape is a powerful tool that integrates various biological databases to provide insights into gene functions, pathways, and interactions. For Gene Ontology (GO) and Kyoto Encyclopedia of Genes and Genomes (KEGG) pathway enrichment analyses, we set the following threshold parameters: *p*-value < 0.01, a minimum overlap of 3 genes, and a minimum enrichment score of 1.5. The GO analysis focuses on the biological processes, cellular components, and molecular functions associated with the DEGs, while the KEGG pathway analysis identifies the signaling pathways and metabolic processes involved.

### 2.5. Determination and Enrichment Analysis of Common Genes

The STRING [14] database (version 12.0)—a widely recognized resource for predicting PPI—was used to identify and analyze common genes associated with PANoptosis. Using the search term ‘PANoptosis’ and the organism ‘Homo sapiens,’ we identified multiple gene sets related to PANoptosis, specifically focusing on those for Homo sapiens by filtering the results. Two PANoptosis-related networks were selected from these results and merged for further analysis.

To investigate the role of PANoptosis-related pathways in COPD and PD, we integrated PANoptosis-associated genes reported in previous studies (PMID:37895022; PMID:37077221) [12,16] with our DEG datasets. Specifically, genes linked to pulmonary functions were intersected with the identified DEGs from COPD and PD cohorts. This intersection ensured that subsequent analyses focused on disease-relevant candidates and allowed for a more comprehensive evaluation of cell death–related mechanisms (pyroptosis, apoptosis, necroptosis) in the context of these conditions. The resulting common genes were then subjected to enrichment analysis using the Metascape database [15]. The enrichment analysis was conducted under strict parameters, with a threshold of *p*-value < 0.01, a minimum overlap of 3 genes, and a minimum enrichment score of 1.5. These parameters were chosen to ensure the robustness and biological relevance of the identified gene sets.

### 2.6. Hub Gene Determination from Protein–Protein Interaction Network

For this study, common genes from DEGs were uploaded to the STRING [14] database to construct a PPI network. To identify significant interactions between coding genes, we applied an interaction confidence score threshold of 0.4. This score indicates the likelihood of a true interaction between two proteins, with higher values representing stronger evidence of interaction.

The resulting PPI network was downloaded and further analyzed using Cytoscape [17] software (version 3.10.3), a powerful platform for visualizing molecular interaction networks. To pinpoint hub genes within the network, we employed the CytoHubba plugin [18] which is designed to hub genes in biological networks. We used several ranking algorithms to prioritize hub genes, including Degree, Edge Percolated Component (EPC), Maximal Clique Centrality (MCC), and Maximum Neighborhood Component (MNC), all of which provide different perspectives on the importance of each gene in the network.

### 2.7. Correlation Analysis of Common Genes Associated with the PANoptosis Pathway

To explore potential interactions among common PANoptosis-related genes in PD and COPD, we conducted a correlation analysis. 78 genes (provided as a Supplementary File) involved in the PANoptosis process were identified from a comprehensive literature search within the STRING database, specifically using the list provided in PMID: 37895022 [12] and PMID:37077221 [16]. Pearson correlation analysis was performed using the Hmisc package (version 5.2.0), where the Pearson correlation coefficient (r) was calculated for each gene pair. Genes exhibiting moderate to strong correlations, defined by a *p*-value < 0.05 and |r| > 0.5, were selected for further analysis. The correlation results were visualized as a heatmap using the ggplot2 package (version 3.5.1) to illustrate the relationships between the common PANoptosis-related genes in both PD and COPD. The heatmap visually represents the strength of correlations between gene pairs, where color intensity indicates the degree of correlation.

### 2.8. Receiver Operating Characteristic Curve Analysis and Determination of Core Genes

To evaluate the potential of PANoptosis-related common genes as biomarkers, we performed ROC curve analysis using the pROC package (version 1.18.5). This analysis quantified the predictive power of each gene by calculating the area under the curve (AUC) values in both PD and COPD datasets.

AUC threshold values were determined based on the empirical distribution of AUC scores across all PANoptosis-related genes. Genes with AUC > 0.73 in PD and AUC > 0.68 in COPD were considered to have acceptable discriminative capacity. These thresholds were selected to balance predictive strength and gene coverage, enabling the inclusion of genes with moderate classification power while preserving biological relevance. Genes meeting the threshold in both diseases were designated as core PANoptosis-related genes.

To further investigate the collective classification utility of these core genes, the datasets were transposed and labeled (1 for disease, 0 for control), and subsequently split into 70% training and 30% testing sets using the caret package (version 6.0–94). A machine learning model was then constructed using the XGBoost package (version 1.7.8.1). The model was trained with the following parameters: objective = “binary:logistic”, eval_metric = “auc”, nrounds = 150, eta = 0.1, max_depth = 3, subsample = 0.8, colsample_bytree = 0.8, gamma = 0, and min_child_weight = 1. These hyperparameters were selected based on common practice in transcriptomics-based classification tasks to provide a balance between model complexity and interpretability. A 5-fold stratified cross-validation was also performed using the xgb.cv() function to evaluate the stability and average performance of the model across folds.

Gene-wise feature importance scores were calculated using the xgb.importance() function. To ensure reproducibility, a fixed random seed was used (set.seed(123)). The ROC curve of the XGBoost model was also plotted to assess its overall classification performance.

### 2.9. Immune Infiltration Analysis

Cell-type Identification by Estimating Relative Subsets of RNA Transcripts (CIBERSORT) algorithm [19] implemented in R, was used to derive immune infiltration matrices from gene expression datasets of PD and COPD. The Wilcoxon rank-sum test was employed to assess differences between the two groups. Subsequently, correlation heatmaps were generated using the corrplot package (version 0.95) to visualize the associations among the 22 immune cell types and the correlations between core genes and immune cells.

### 2.10. Analysis of Drug–Gene Connection

Drug Gene Interaction Database (DGIDB) [20], a comprehensive database of drug–gene interactions, was used to identify potential drugs targeting the common genes of interest. The common genes were uploaded to the DGIDB platform for analysis. Drugs with an interaction score exceeding 2.5 were considered as potentially adequate candidates.

## 3. Results

### 3.1. Data Preparation

**Figure 2:**
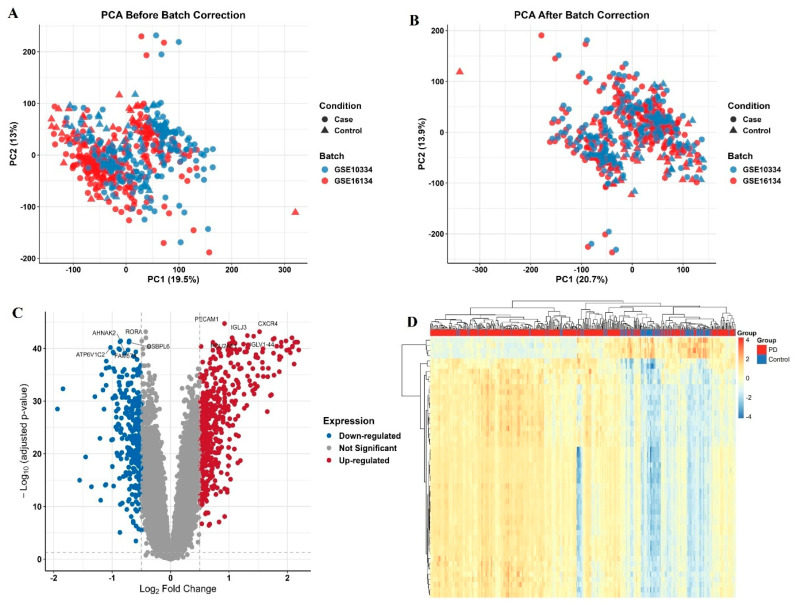
Principal component analysis (PCA), volcano plot, and hierarchical clustering heatmap of differentially expressed genes (DEGs) in Periodontitis (PD) samples. (**A**) PCA plot before batch correction showing batch-driven clustering. (**B**) PCA plot after batch correction showing improved sample integration and separation by condition. (**C**) Volcano plot of DEGs between PD and control groups. Red: up-regulated, blue: down-regulated genes (adj. *p* < 0.05, |log2 FC| > 0.5). Top genes are labeled. (**D**) Heatmap of the top 50 DEGs by fold change. Samples cluster distinctly by group (PD vs. Control).

**Figure 3:**
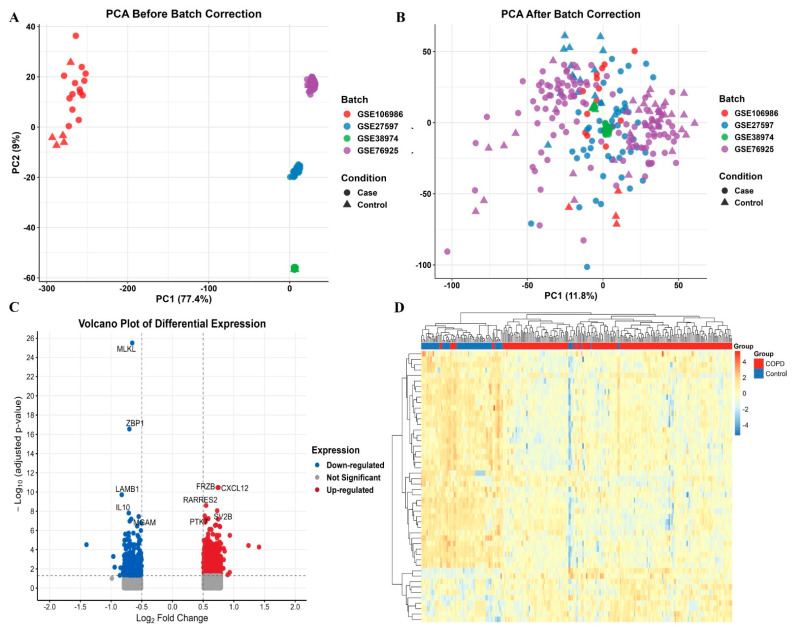
Principal Component Analysis (PCA), differential expression, and hierarchical clustering of COPD and control samples. (**A**) PCA plot before batch effect correction showing clear dataset-based separation among samples from different GEO datasets. (**B**) PCA plot after batch effect correction, where case and control samples are more homogenously distributed across batches. (**C**) Volcano plot of differentially expressed genes. Red points indicate up-regulated genes, blue points indicate down-regulated genes, and grey points are not significantly different. Thresholds applied: adjusted *p*-value < 0.05 and |log2 FC| > 0.5). (**D**) Heatmap of the top 50 DEGs by fold change. Samples cluster distinctly by group (COPD vs. Control).

Following batch correction, PD datasets (GSE16134, GSE10334) and COPD datasets (GSE27597, GSE38974, GSE76925, and GSE106986) were harmonized for analysis. The PD cohort included 424 cases and 113 controls, while the COPD cohort comprised 196 cases and 70 controls. All datasets were based on microarray platforms to ensure consistency. Batch effects were corrected using the COMBAT method, and subsequent PCA confirmed successful dataset integration with minimal residual technical variation (Figure 2 and Figure 3). Moreover, similar conclusions were also observed in a recent study [21], which used only microarray-based PD datasets and identified overlapping gene signatures.

### 3.2. Functional Enrichment Analysis of Differentially Expressed Genes

After batch correction and merging of PD datasets (GSE16134, GSE10334) and COPD datasets (GSE27597, GSE38974, GSE76925, GSE106986), differential expression analysis was performed using the limma package (adjusted *p* < 0.05, |log2 FC| > 0.5). This yielded 488 downregulated and 314 upregulated genes in PD, and 723 upregulated and 324 downregulated genes in COPD (Figure 2C and Figure 3C). The complete gene lists are provided in Supplementary Tables S1 (PD) and S2 (COPD).

**Figure 4:**
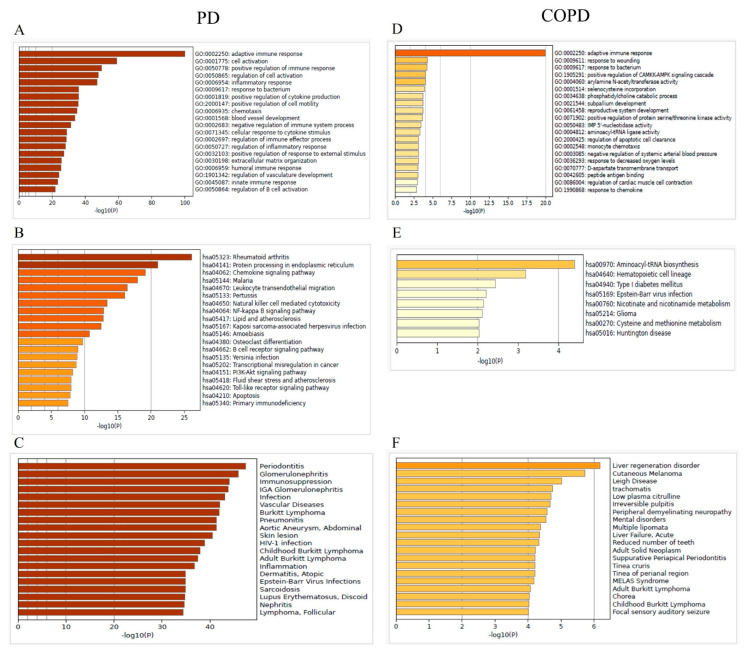
Summary of enrichment analysis in PD and COPD. (**A**) and (**D**), Gene Ontology (GO); (**B**) and (**E**), Kyoto Encyclopedia of Genes and Genomes (KEGG); and (**C**) and (**F**), DisGeNET.

Functional enrichment analysis using GO and KEGG pathways, along with Disease Gene Network (DisGeNET), revealed that the DEGs in both PD and COPD were associated with pathways such as “adaptive immune response” and “response to bacterium” (Figure 4).

### 3.3. Identification of PANoptosis-Related Core Genes and Functional Enrichment of Hub Genes

**Figure 5:**
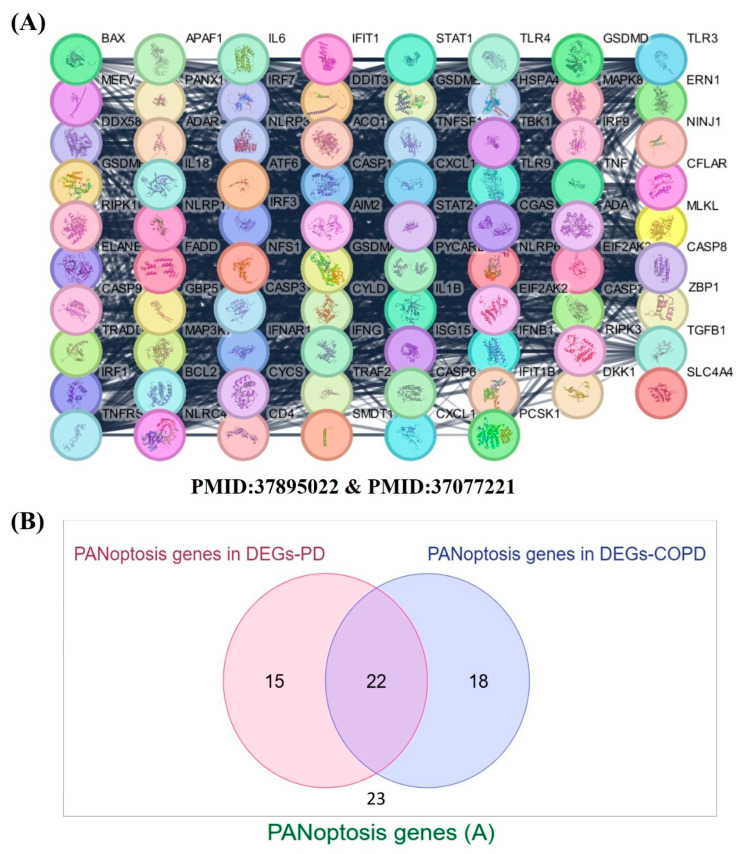
(**A**) STRING-based protein–protein interaction (PPI) network of 78 curated PANoptosis-related genes (PMID: 37895022; PMID: 37077221), showing functional crosstalk between apoptosis, pyroptosis, and necroptosis regulators. A list of the genes is provided as a Supplementary File (Supplementary Table S3). (**B**) Venn diagram showing the overlap between PANoptosis genes and DEGs in PD and COPD. Of the 78 curated genes, 15 were DEGs only in PD, 18 only in COPD, 22 shared across both datasets, and 23 were not differentially expressed. ‘The PANoptosis genes (A)’ depicted in panel (**B**) correspond to the list of 78 PANoptosis-related genes referenced in panel (**A**).

A reference set of 78 PANoptosis-related genes was curated from recent literature (PMID: 37895022; PMID: 37077221). Figure 5A illustrates their STRING-based protein–protein interaction (PPI) network, highlighting functional associations and hubs mediating crosstalk between apoptosis, pyroptosis, and necroptosis. Next, we intersected this gene set with the DEGs identified in PD (802 genes) and COPD (1047 genes). This analysis showed that: 15 PANoptosis genes were DEGs only in PD, 18 PANoptosis genes were DEGs only in COPD, 22 PANoptosis genes were shared DEGs across both datasets, and the remaining 23 PANoptosis genes were not differentially expressed in either disease. Thus, Figure 5B demonstrates the overlap of DEGs with PANoptosis regulators, highlighting 22 shared PANoptosis-related genes as potential molecular signatures underlying both PD and COPD.

**Figure 6:**
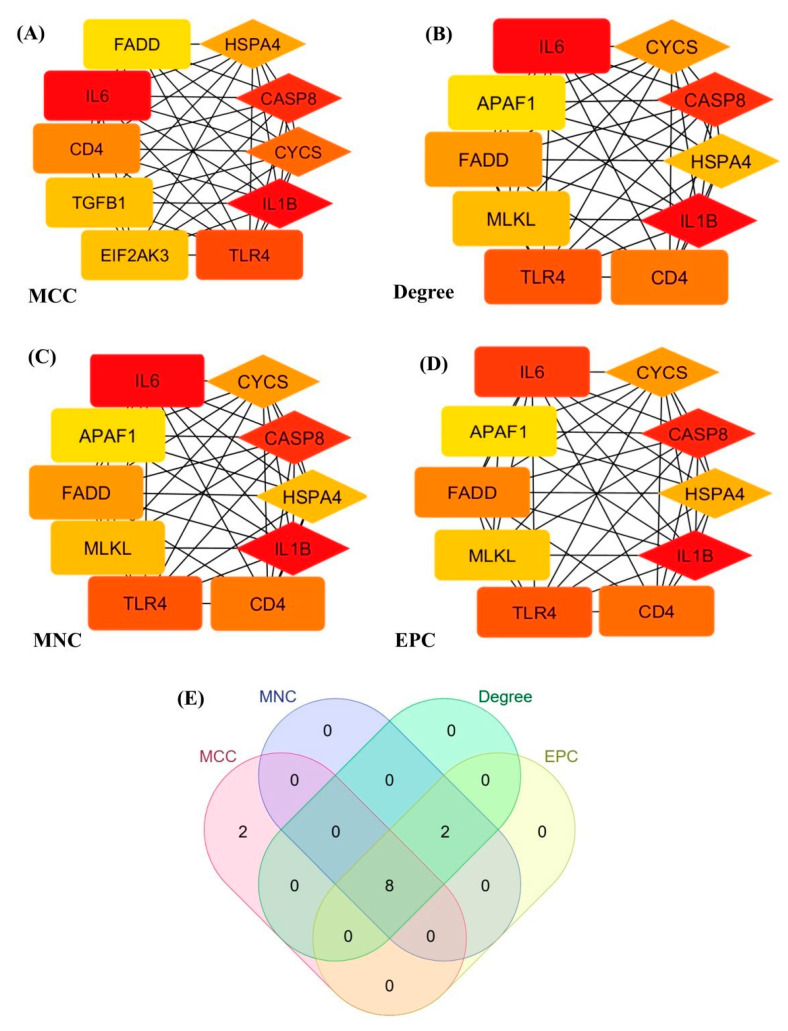
Key common genes were identified using multiple ranking methods. Panels (**A**–**D**) illustrate the top 10 genes identified in protein–protein interaction (PPI) networks based on MCC, Degree, MNC, and EPC scoring metrics, respectively. The analysis revealed that the top 10 genes across these four ranking methods include *CASP8*, *CD4*, *CYCS*, *FADD*, *HSPA4*, *IL1B*, *IL6*, and *TLR4*. Four of them were core genes. (**E**) Venn diagram summarizing the overlap of the top-ranked hub genes identified by the four cytoHubba metrics (MCC, Degree, MNC, and EPC); numbers indicate the count of genes shared by the corresponding method(s), with the central intersection representing genes common to all four.

Figure 6 presents the topological properties of the PANoptosis-related hub gene network, identifying highly connected nodes with significant regulatory roles. The analysis revealed that the top 10 genes across four different ranking methods include *CASP8*, *CD4*, *CYCS*, *FADD*, *HSPA4*, *IL1B*, *IL6*, and *TLR4*, with four of them being core genes. These key hub genes were enriched in inflammatory and immune-related pathways, reinforcing their potential involvement in PANoptosis-driven tissue damage and disease progression.

**Figure 7:**
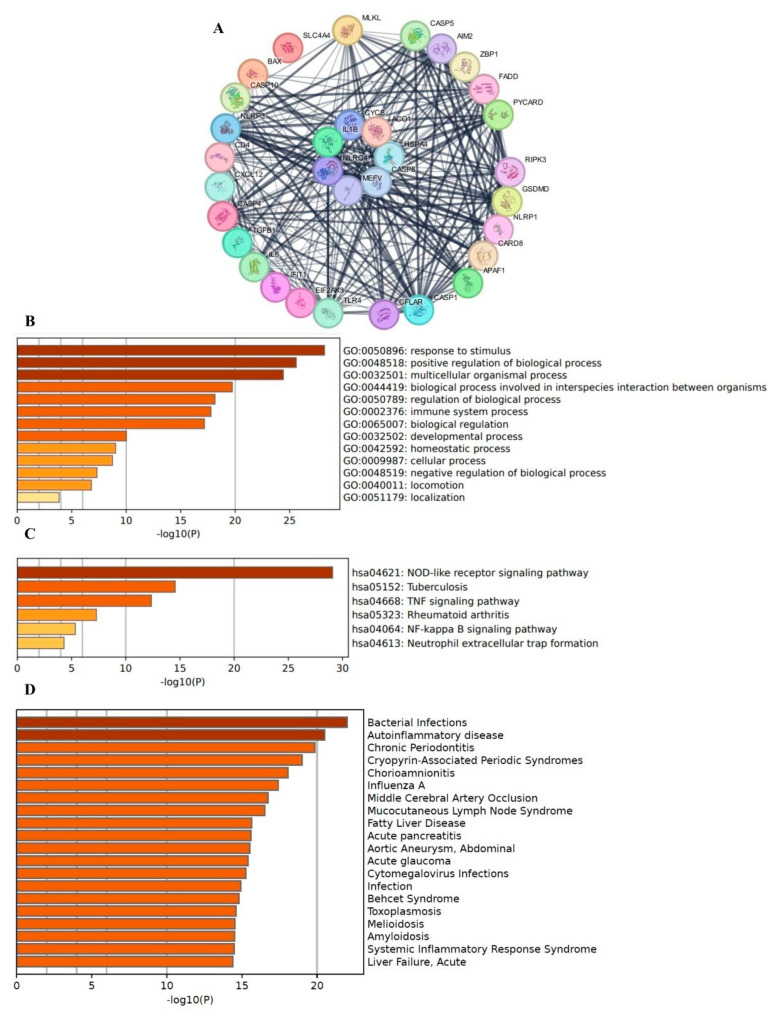
Cross-talk PANoptosis-related genes in PD and COPD were constructed using Cytoscape. (**A**) confidence scale cutoff of 0.4 was applied in the STRING database, with a maximum of 10 additional interactors. Summary of enrichment analysis in PD and COPD. (**B**) Gene Ontology (GO), (**C**) Kyoto Encyclopedia of Genes and Genomes (KEGG) and (**D**) DisGeNET.

Additionally, functional enrichment analyses were performed to explore the biological significance of the identified hub genes (Figure 7A). GO analysis (Figure 7B) highlighted key biological processes associated with inflammatory response, immune regulation, and apoptotic signaling, indicating the involvement of PANoptosis-related genes in disease progression. KEGG pathway analysis (Figure 7C) revealed significant enrichment in inflammatory and infection-related pathways such as nucleotide-binding oligomerization domain -like receptor (NLR) signaling, Tumor Necrosis Factor (TNF) signaling, Nuclear Factor kappa-light-chain-enhancer of activated B cells (NF-kappa B) signaling and Rheumatoid artritis, reinforcing the role of PANoptosis in chronic inflammatory disorders. DisGeNET analysis (Figure 7D) further confirmed the association of these genes with various inflammatory and immune-mediated diseases including bacterial infections, autoinflammatory, chronic periodontitis, influenza A, supporting their potential as biomarkers or therapeutic targets. 

### 3.4. Correlation Analysis of Common Genes Associated with the PANoptosis Pathway

**Figure 8:**
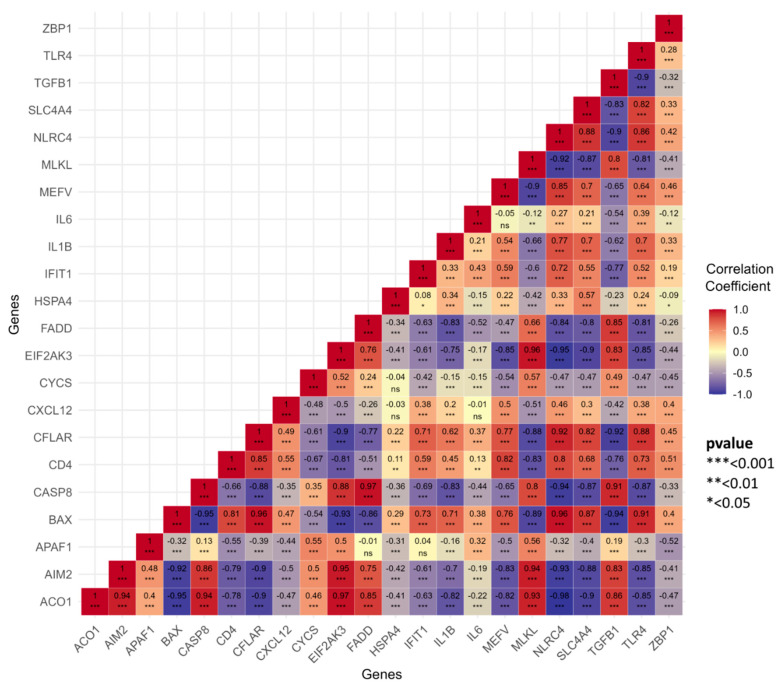
Heatmap of correlation and significance for gene expression (PD and COPD). ns: not significant (*p* > 0.05).

To investigate the interactions between common genes involved in the PANoptosis pathway in PD and COPD, a heatmap was generated to visualize the correlation and statistical analysis of expression profiles for 22 PANoptosis-related genes in both diseases (Figure 8). The heatmap displays Pearson correlation coefficients between gene pairs, providing insight into the strength and direction of their relationships. Statistical significance is indicated by asterisks: *** (*p* < 0.001), ** (*p* < 0.01), and * (*p* < 0.05).

Pearson correlation coefficients were calculated to assess the relationships between common PANoptosis-related genes in PD and COPD separately. The intensity of the color squares in the heatmap reflects the strength of the correlation, with red representing positive correlations and blue indicating negative correlations. Darker shades correspond to stronger correlations, highlighting the most significant associations (Figure 8).

### 3.5. Identification of Common Core Genes Related-PANoptosis by ROC Curve Analysis

**Figure 9:**
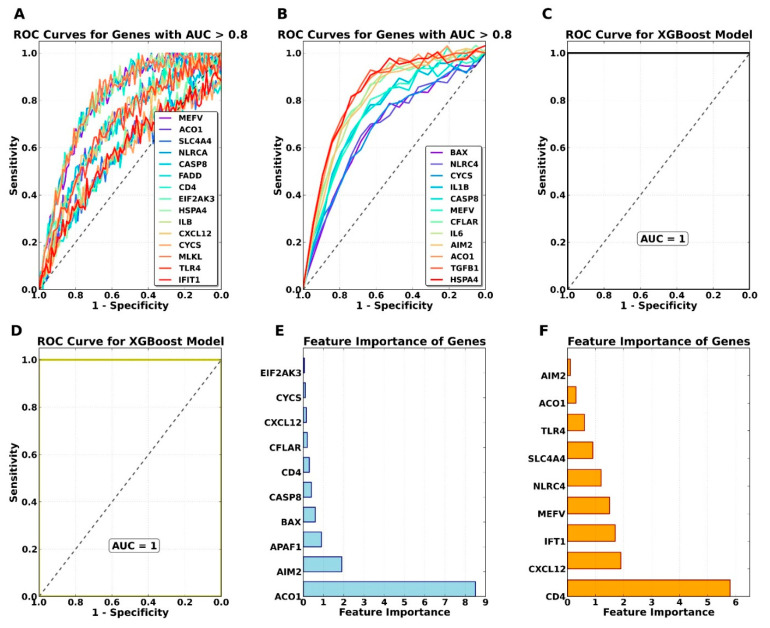
(**A**) ROC curve analysis showcasing the performance of common PANoptosis genes in PD samples; (**B**) ROC curve analysis illustrating the performance of common PANoptosis genes in COPD samples; (**C**) ROC curve for XGBoost in PD samples, achieving an AUC of 1.0; (**D**) ROC curve for XGBoost in COPD samples, with an AUC of 1.0; (**E**) Feature importance analysis for PANoptosis-related genes in PD samples; (**F**) Feature importance analysis for PANoptosis-related genes in COPD samples.

The intersection of common PANoptosis-related genes identified seven key genes: *MEFV*, *ACO1*, *NLRC4*, *CASP8*, *HSPA4*, *IL1B*, and *CYCS*. These genes represent a critical link between cross-talk mechanisms and the PANoptosis pathway, with an AUC value greater than 0.8, indicating strong diagnostic potential. ROC curve analysis was conducted to evaluate the diagnostic capability of these genes (Figure 9). Based on these findings, *MEFV*, *ACO1*, *NLRC4*, *CASP8*, *HSPA4*, *IL1B*, and *CYCS* were identified as core genes shared between PD and COPD. To further evaluate the collective impact of these core genes on both diseases, a classification model was developed using XGBoost. The model demonstrated perfect classification efficiency, achieving an accuracy of 1.0 for both PD and COPD (Figure 9). These results underscore the pivotal role of the core genes in the onset and progression of PD and COPD, highlighting significant interactions among these genes and their potential as diagnostic biomarkers. These seven core genes were selected based on a multi-tiered strategy: they were (1) significantly differentially expressed in both PD and COPD datasets, (2) previously reported as PANoptosis-related in the literature (PMID: 37895022 and 37077221), and (3) exhibited strong diagnostic power, defined as having AUC > 0.80 in both PD and COPD. This stringent selection ensured that only genes with biological relevance and robust classification ability across both diseases were included.

### 3.6. Immune Infiltration

**Figure 10:**
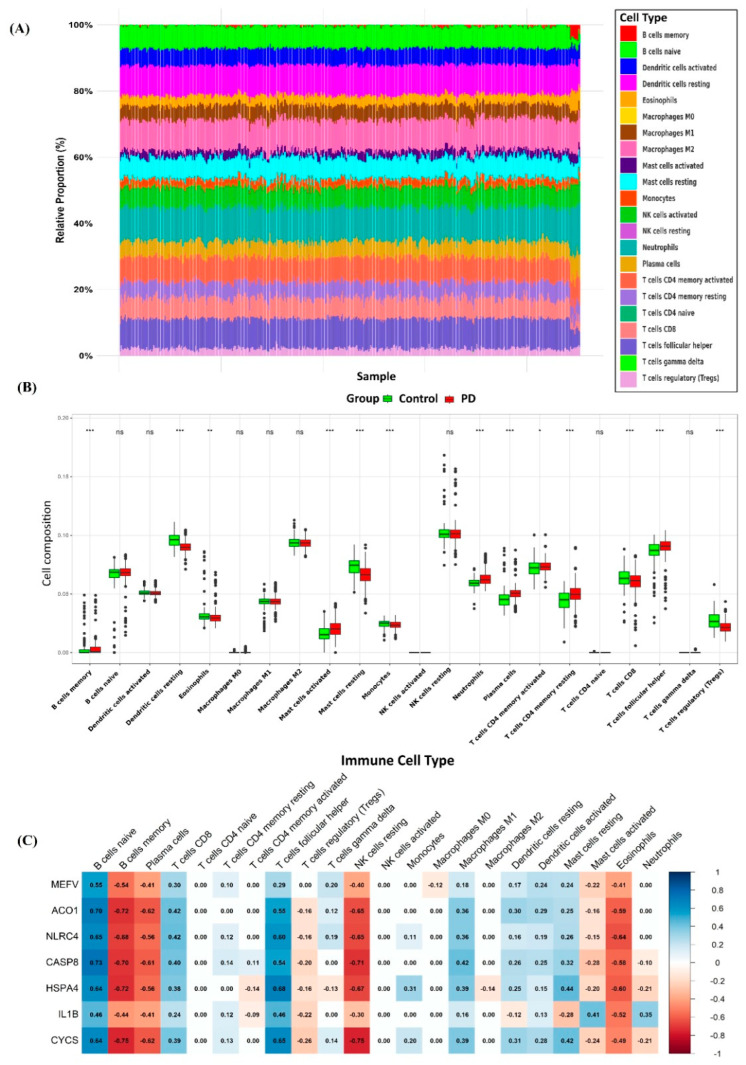
(**A**) Immune cell infiltration profiles for each sample in PD. (**B**) Boxplots depicting the expression levels of individual immune cell types in PD, with * *p*-value < 0.05, ** *p*-value < 0.01, and *** *p*-value < 0.001. ns: not significant (*p* > 0.05). (**C**) Correlation analysis between core genes and immune cell populations in PD.

**Figure 11:**
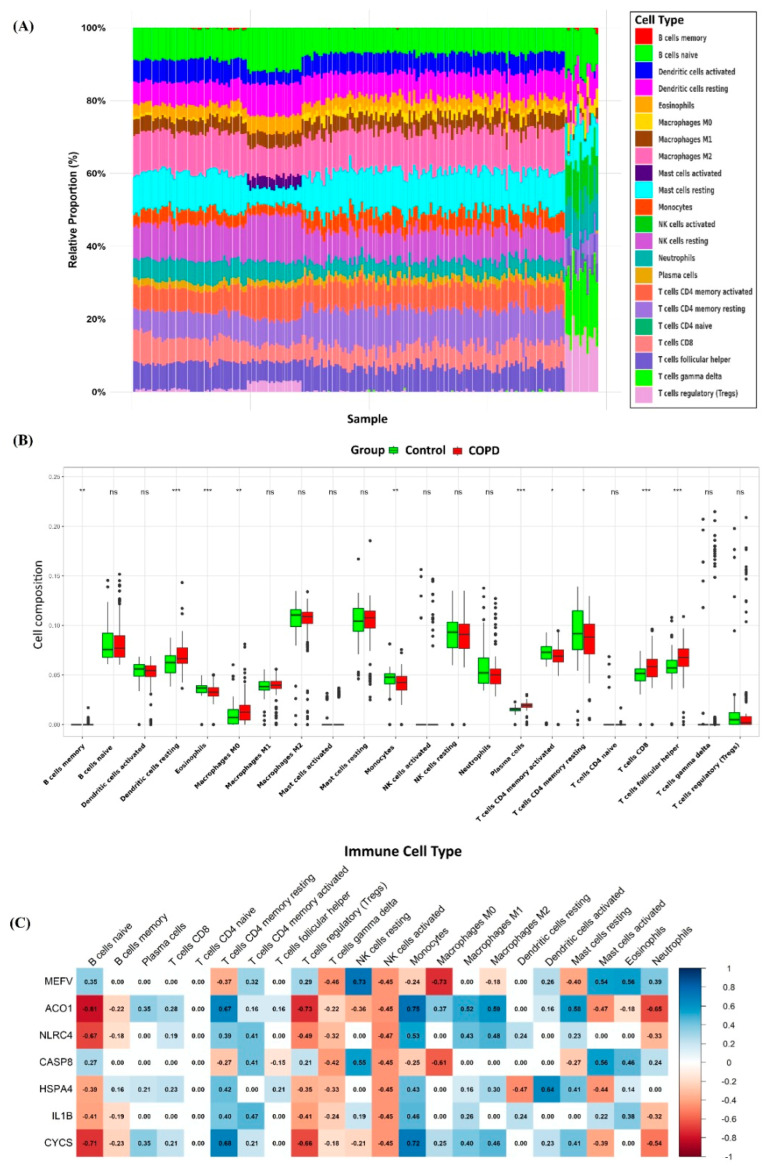
(**A**) Immune cell infiltration profiles for each sample in COPD. (**B**) Boxplots depicting the expression levels of individual immune cell types in COPD, with * *p*-value < 0.05, ** *p*-value < 0.01, and *** *p*-value < 0.001. ns: not significant (*p* > 0.05). (**C**) Correlation analysis between core genes and immune cell populations in PD.

Based on a meta-analysis examining the relationship between PD and respiratory diseases, we identified a profound interplay between PD, COPD, and immune system dysregulation. To explore the role of immune cells in these diseases and to assess the regulatory influence of key genes, we performed an extensive immune infiltration analysis using the CIBERSORTx algorithm applied to datasets from both PD and COPD. The analysis revealed statistically significant differences (*p* < 0.05) across 22 immune cell subpopulations when comparing diseased tissues from PD and COPD to their corresponding controls. Based on the statistical annotations (*, **, ***) shown in Figure 10B and Figure 11B, a subset of immune cell types demonstrated significant changes in infiltration levels across both disease models. A total of nine immune cell types were found to be significantly altered (*p* < 0.05) in both PD and COPD cohorts: B cells memory, dendritic cells resting, eosinophils, monocytes, plasma cells, CD4 memory T cells (activated and resting), CD8 T cells, and follicular helper T cells. This consistent immune infiltration pattern highlights shared immune dysregulation across both diseases and may suggest the involvement of PANoptosis-linked pathways.

As shown in the Pearson correlation heatmaps for COPD and PD (Figure 7), the correlation analysis between seven core genes and immune cell subpopulations reveals both distinct and overlapping immune patterns in the two diseases (Figure 10 and Figure 11). In both conditions, core genes such as *MEFV*, *ACO1*, *NLRC4*, *CASP8*, *HSPA4*, *IL1B*, and *CYCS* exhibit varying degrees of correlation with immune cell subsets.

### 3.7. Drug–Gene Interaction Analysis

**Figure 12:**
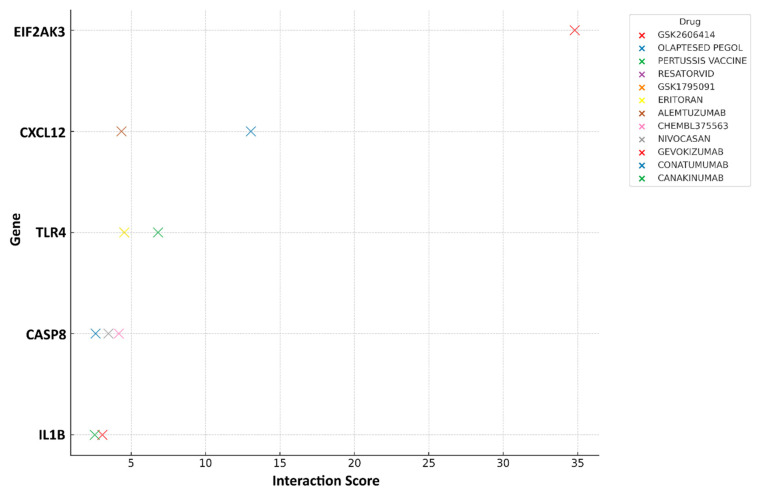
The plot showing the interaction scores of various drugs with specific genes. Genes are displayed on the *y*-axis, while interaction scores are plotted along the *x*-axis. Each drug is represented by a distinct color, illustrating its interaction with one or more genes. Note that the interaction scores of Resatorvid, Eritoran, GSK1795091, and Alemtuzumab range between 4.35 and 4.54, which causes their markers to overlap on the chart.

Based on the common PANoptosis genes, we identified 12 potential therapeutic drugs for COPD patients with PD (Figure 12). Two drugs are correlated with *CXL12*, four are linked to *TLR4*, three are associated with CASP8, two are associated with *IL1B*, and a drug with the highest score is related to *EIF2AK3*.

## 4. Discussion

This study aims to investigate the role of PANoptosis, a novel form of regulated cell death, in the pathophysiology of both PD and COPD, exploring potential shared gene signatures and their implications. By focusing on PANoptosis, we seek to uncover common molecular mechanisms that may contribute to the development and progression of these two inflammatory diseases. In our bioinformatics analysis, we identified seven core genes—*MEFV*, *NLRC4*, *CASP8*, *IL1B*, *ACO1*, *CYCS*, and *HSPA4*—that are involved in critical inflammatory pathways. In the following sections, we examine their individual roles and how they may collectively contribute to the shared pathogenesis of PD and COPD.

The *MEFV* gene encodes Pyrin, a protein that is central to the formation of the Pyrin inflammasome. Upon activation by pathogen-associated molecular patterns (PAMPs) or inflammatory signals, Pyrin induces the activation of caspase-1, which in turn processes and releases IL-1β and IL-18, key pro-inflammatory cytokines [22]. Its dysregulation contributes to autoinflammatory syndromes, including familial Mediterranean fever (FMF) and other conditions such as hyper IgD syndrome and pyogenic arthritis [22]. Variations in the *MEFV* gene have been identified as a potential link between FMF and PD, suggesting a strong genetic and inflammatory connection that may contribute to increased susceptibility and severity of PD [23]. No studies have been found that directly investigate the mechanistic relationship or clinical significance of *MEFV* gene mutations in COPD. However, the B30.2 domain of Pyrin can also inhibit caspase-1 activity, preventing excessive IL-1β production and curbing inflammation. Mutations in *MEFV* can disrupt this regulatory mechanism, leading to an overproduction of IL-1β and contributing to chronic inflammation [22]. Our study highlights *IL-1β* and *MEFV* as central hub genes, supporting their role in linking PD and COPD. Moreover, the identification of IL-1β inhibitors such as canakinumab and gevokizumab suggests potential therapeutic strategies for managing both diseases [24].

Neuronal apoptosis inhibitory proteins (NAIPs) are cytosolic receptors that detect bacterial proteins and recruit NLR family CARD domain-containing protein 4 (NLRC4) to form inflammasomes. When activated by bacterial ligands such as flagellin, NLRC4 triggers immune responses by promoting the release of inflammatory cytokines, including IL-1β and IL-18, and facilitating the expulsion of infected cells to limit bacterial spread [25]. ASC (contains pyrin domain) is needed to fully process pro-IL-1β in this pathway. It bridges NAIPs to caspase-1, activating the inflammasome and triggering the release of pro-inflammatory cytokines like IL-1β [26]. Our KEGG analysis highlighted the NLR signaling pathway, underscoring NLRC4’s involvement in inflammation shared by PD and COPD. In the gut, the NAIP-NLRC4 inflammasome helps expel infected epithelial cells, but both caspase-1 and caspase-8 are required for this process [25]. Caspase-8 plays a crucial role in regulating apoptosis within innate immunity, and its absence or inhibition can trigger necroptosis [27]. The interaction between NLRC4, ASC, and caspase-8 highlights the shared inflammatory pathway between PD and COPD, where IL-1β and caspase-8 activation play central roles. No clinical study has specifically explored the link between *NLRC4* and COPD. However, a study has investigated the relationship between NLRC4 and PD, supporting the connection between the NLRC4 inflammasome and periodontal inflammation [28]. This suggests that targeting the NLRC4-IL-1β pathway could be a potential therapeutic approach for managing periodontal diseases. A meta-analysis shows a significant link between *IL-1β* gene polymorphisms and an increased risk of PD [29]. Another meta-analysis reveals a complex relationship between the *IL-1β*-31T/C polymorphism and COPD, where heterozygous individuals have an increased risk, while homozygous individuals may be protected from COPD [30]. These two meta-analyses further emphasize the importance of *IL-1β* in both diseases. These suggests that both diseases involve complex inflammasome-driven inflammation, not just a single model.

The *ACO1* gene, also known as Aconitase or IRP1, encodes a bifunctional protein crucial for both intracellular iron regulation and the TCA cycle. It interacts with ferritin and transferrin receptor mRNA to maintain iron homeostasis [31], whereas IREB2 (IRP2) is an IRE-binding protein with no aconitase function. Disruption of both IRP1 and IRP2 impairs neutrophil development and differentiation in the bone marrow, yielding immature neutrophils with abnormally high glycolytic and autophagic activity, resulting in neutropenia [32]. Elevated iron levels in the lungs, particularly in smokers, can contribute to inflammation and COPD development. IREB2 has been identified as a susceptibility gene for COPD, while our study highlights *ACO1*’s role in this pathway [33]. Iron metabolism is also disrupted in PD, with a meta-analysis showing lowered hemoglobin levels and iron imbalance, leading to anemia of inflammation. Transferrin, which carries iron, is affected in periodontal disease. Some bacteria like *Prevotella intermedia*, *Campylobacter rectus*, and *Porphyromonas gingivalis* can take iron from transferrin or break it down, releasing free iron that can damage tissues. Ferritin secretion, influenced by cytokines like IL-1 and TNF-α, is also linked to periodontal inflammation [34]. Given these connections, *ACO1* may play a shared role in the pathogenesis of both periodontal disease and COPD through its regulation of iron metabolism.

*HSPA4*, a member of the heat shock protein 70 (HSP70) family, plays a critical role in maintaining protein homeostasis under stress conditions. *HSPA4* expression is reduced during inflammation, which can lead to the activation of the NF-κB pathway, a central regulator of inflammatory responses [35]. Its overexpression is linked to increased cell survival, metastasis, and resistance to apoptosis, serving as a potential prognostic marker in cancers. It also regulates immune cells such as macrophages, dendritic cells, and NK cells, affecting their ability to detect and destroy cancer cells [36]. Since inflammation is central to the progression of both PD and COPD, reduced *HSPA4* expression in inflamed tissues may exacerbate disease severity [37,38]. Our KEGG analysis also pointed to the NF-κB signaling pathway as crucial for the pathogenesis of PD and COPD, further supporting indirect role of *HSPA4* in modulating the inflammatory process. There is currently no clinical study available that has investigated the relationship between *HSPA4* and either PD or COPD.

Cytochrome c, or *CYCS*, is a mitochondrial protein involved in oxidative phosphorylation and apoptosis. When a cell is damaged, cytochrome c can be released into the extracellular space, acting as a Damage-Associated Molecular Pattern (DAMP). While it normally induces non-inflammatory apoptosis in mitochondria, outside the cell it can trigger inflammation and signal severe mitochondrial damage, activating immune responses [39]. Both PD and COPD are associated with oxidative stress and cell death [40]. In COPD, the release of cytochrome c from mitochondria triggers apoptosis, contributing to tissue damage in the lungs [41]. Clinical studies show elevated cytochrome c levels in COPD patients, particularly in skeletal and respiratory muscles. Higher plasma levels are associated with worse lung function and symptoms, but not with inflammation or smoking history, suggesting cytochrome c as a potential COPD biomarker [42,43]. Similarly, in PD, oxidative stress and cell death play significant roles in disease progression [44]. The involvement of *CYCS* in both oxidative stress and apoptosis mechanisms suggests it may contribute to the pathogenesis of both PD and COPD, linking mitochondrial dysfunction to the inflammatory processes observed in these diseases.

To enhance the mechanistic perspective, the identified PANoptosis-related genes were mapped to key signaling pathways. MEFV and NLRC4 are involved in inflammasome pathways (pyrin and NLRC4, respectively), while CASP8 regulates apoptosis and necroptosis via caspase signaling. IL1B is a downstream effector of the NLRP3 inflammasome, central to pyroptosis. ACO1 is linked to iron metabolism and oxidative stress, CYCS to mitochondrial apoptosis and oxidative phosphorylation, and HSPA4 to cellular stress responses. These associations support the role of PANoptosis-related processes in the shared pathogenesis of PD and COPD.

This study explores the immune cell infiltration landscape in PD and COPD, highlighting shared immune features and disease-specific differences. The immune cell composition in both conditions shows significant overlap but also key distinctions, especially in memory B cells, dendritic cells, eosinophils, monocytes, plasma cells, CD4+ and CD8+ T cells, and T follicular helper (Tfh) cells.

CD4+ T cells play various roles in immune response, including supporting CD8+ T cell expansion, regulating inflammation, promoting tissue homeostasis, and aiding in wound healing. Memory CD4+ T cells provide long-term protection by offering a quicker response to reinfections [45]. In our study, we found lower levels of both activated and resting memory CD4+ T cells in PD and COPD patients compared to healthy individuals. CD8+ T cells prevent excessive immune activation and tissue damage [46]. In line with our findings, a meta-analysis reported increased CD4+ T cells and decreased CD8+ T cells in PD patients [47]. In COPD, both CD8+ and CD4+ T cells accumulate in lung tissues, contributing to airway inflammation. CD8+ T cells damage lung tissue directly, while CD4+ T cells regulate inflammation through cytokine release [48]. In our study, both CD8+ and CD4+ T cells increased in the disease state of COPD. Follicular helper T cells (Tfh), a subset of CD4+ T cells, regulate B cell immunity by promoting B cell proliferation, immunoglobulin class switching, and antibody maturation. In PD, gingival tissues show signs of Tfh cell involvement, supporting B cell activation and antibody responses to control periodontal lesions [49]. In COPD, disease severity is linked to the development of tertiary lymphoid organs (TLOs). TLO formation is rare in healthy individuals but significantly increases in COPD patients, particularly in advanced stages (GOLD III/IV). The presence of Tfh-like cells in lung TLOs suggests that Tfh cells contribute to their formation and maintenance [50].

Memory B cells play a crucial role in the inflammatory response of both PD and COPD. In PD, they are strategically positioned near the junctional epithelium, influencing local immune responses and maintaining tissue homeostasis [46]. Similarly, in COPD, memory B cells contribute to chronic inflammation, promoting antibody production that exacerbates lung inflammation [48]. Plasma cells, derived from B lymphocytes, are known for producing antibodies but have also been identified as significant cytokine producers, playing a key role in immune regulation in autoimmune and infectious diseases. In PD, plasma cells and B cells, initially a minor component in early lesions, become more prominent as the disease progresses, contributing significantly to advanced lesions [46,51]. In COPD, B cell activity increases, and upon activation, they differentiate into plasma cells that produce large quantities of antibodies, further exacerbating inflammation and oxidative stress [48].

Classical monocytes are short-lived cells that differentiate into macrophages and dendritic cells, playing a key role in inflammation by recognizing pathogens, secreting cytokines, and recruiting other immune cells. A decrease in classical monocytes in PD suggests enhanced inflammation within the tissue, as confirmed in our study. Nonclassical monocytes, which patrol the vascular endothelium, help maintain vascular homeostasis by clearing dying endothelial cells. In PD, endothelial dysfunction is observed, and changes in nonclassical monocytes may contribute to vascular issues [47]. In COPD, endothelial dysfunction is also present, affecting pulmonary vasculature and disease severity [52]. Altered nonclassical monocyte function in PD could exacerbate endothelial dysfunction, ref. [47] potentially impacting COPD progression.

Dendritic cells are key antigen-presenting cells that help prime the immune system. In periodontal lesions, dendritic cells contribute to pro-inflammatory cytokine production, which exacerbates inflammation and tissue damage in PD [53]. CIBERSORT results from a previous study showed a significant decrease in memory B cells and resting dendritic cells in periodontitis [54]. However, our study found memory B cells to be more abundant in PD, while resting dendritic cells were more prevalent in healthy conditions. Resting dendritic cells are crucial for immune homeostasis in the lungs, regulating the balance between tolerance and immunity to prevent excessive inflammation in COPD [50]. In our study, resting dendritic cells decreased in PD but increased in COPD during disease progression.

Eosinophils are mainly known for their role in allergic reactions and parasite elimination. During infection or inflammation, the number of circulating eosinophils decreases, which may be due to the release of chemotactic factors that lead to their accumulation in tissues. Eosinophils also act as antigen-presenting cells and can recognize Gram-negative bacteria, releasing proteins that contribute to inflammation [55]. In our study, we found higher eosinophil counts in healthy individuals compared to those with PD. A recent bioinformatics study on PD and immune cells did not identify eosinophils, as it used 21 immune cell types instead of 22. Furthermore, only one of the two gingival tissue-based databases from our study was included in their analysis [53]. Eosinophilic COPD is associated with eosinophil-driven airway inflammation, which leads to more frequent exacerbations and distinct treatment responses. Eosinophils release inflammatory mediators that contribute to airway narrowing, excessive mucus production, and airway remodeling, worsening the condition [56]. Targeting eosinophil-driven inflammation pathways may offer therapeutic benefits for both COPD and PD.

In this study, we identified 12 potential drugs targeting common genes associated with both PD and COPD, all with interaction scores of at least 2.5. Notably, GSK2606414 (EIF2AK3) has been tested in vitro for PD [57], showing promise, though further research is needed. In chronic diseases, endoplasmic reticulum (ER) stress and inflammation reinforce each other, sustaining inflammation. Targeting Protein kinase RNA-like Endoplasmic Reticulum Kinase (PERK), a key ER stress mediator, offers a promising strategy for selective immunoregulation without affecting normal immune function. The PERK inhibitor GSK2606414 supports this finding by reducing pathological inflammation while preserving immune homeostasis [58]. Although it has not been evaluated in clinical trials for COPD, a mechanistic in vitro study simulating COPD pathology through efferocytosis demonstrated that ER stress suppresses efferocytosis in murine alveolar macrophages. Notably, treatment with GSK2606414 successfully restored this process, suggesting its therapeutic potential in COPD [59].

Canakinumab, an IL-1β monoclonal antibody, has emerged as a potential therapeutic option for both PD and COPD due to its ability to modulate the inflammatory processes common to both diseases [24,60,61,62]. It can cause side effects, including severe infections, a decreased ability of the body to fight infections due to immunosuppression, serious allergic reactions, and an increased risk of infection if taken with live vaccines [63]. While no clinical trials have been conducted to evaluate its efficacy in PD, a completed clinical trial (NCT00581945) assessed its safety and efficacy in COPD patients. However, no results or peer-reviewed publications from this study have been made publicly available to date. Gevokizumab, an IL-1β-targeting monoclonal antibody, shows potential as a therapeutic candidate for both PD and COPD by modulating inflammation. It functions by binding to IL-1β and preventing its interaction with the IL-1 receptor type I, thereby inhibiting downstream inflammatory signaling [64]. Reported side effects affect various systems, including infections, hypertension, gastrointestinal disorders, blood disorders, muscle pain, neurological, ocular, respiratory, and skin reactions [63]. Despite its therapeutic promise, no clinical trials or in vitro studies have yet explored its application specifically in PD or COPD, highlighting a gap and an opportunity for future research.

Olaptesed pegol modulates the immune system by inhibiting CXCL12. In chronic lymphocytic leukemia, this inhibition mobilizes leukemia cells into the circulation and prevents their migration to protective niches [65]. In solid tumors, CXCL12 inhibition reduces the recruitment of CD68^+^ immunosuppressive macrophages induced by anti-VEGF therapy. This helps restore antitumor immune responses and enhances treatment efficacy by limiting immune cell infiltration into hypoxic tumor sites [66]. The most frequently reported adverse events in clinical trials were injection site reaction, gastrointestinal disorders such as diarrhea and nausea, and hematological changes [67]. No clinical or in vitro studies have been identified investigating the effects of olaptesed pegol in relation to PD or COPD. Alemtuzumab, an FDA-approved IgG1 monoclonal antibody against the CD52 antigen. CXCL12 is a chemokine that regulates key processes like embryogenesis, blood cell formation, angiogenesis, and inflammation by guiding the movement and activation of stem cells, immune cells, and endothelial cells, mainly via its receptor CXCR4 [68]. Blocking the CXCL12–CXCR4 pathway disrupts cell migration, weakens tumor-stroma interactions, reduces angiogenesis, and boosts immune responses. This can make tumor cells more sensitive to treatment. In particular, combining CXCR4 blockers with alemtuzumab may improve its effectiveness by making tumor cells easier to target and helping immune cells reach the tumor [69]. Alemtuzumab induces immune alterations such as cytokine-release syndrome, increased viral infection risk, and secondary autoimmunity due to aberrant T and B cell recovery that underlie many of its adverse effects [70]. No clinical studies have been identified investigating the use of alemtuzumab in the context of PD or COPD.

Additionally, we identified four drugs targeting Toll-like receptor 4 (TLR4), a critical component of the innate immune response predominantly expressed on monocytes, macrophages, and dendritic cells. TLR4 recognizes PAMP such as lipopolysaccharides (LPS), playing a significant role in inflammatory signaling. Eritoran, a synthetic lipid A analogue, functions as a TLR4 antagonist by binding to the TLR4–MD-2 complex, thereby inhibiting downstream inflammatory signaling. This helps decrease the production of inflammation-causing substances like TNF-α and IL-6 [71]. Similarly, Resatorvid is a small-molecule inhibitor that blocks TLR4-mediated cytokine production by targeting the receptor’s intracellular domain [72]. In contrast, GSK1795091 acts as a TLR4 agonist [73]. Preclinical study show antitumor effects, especially when combined with other immunotherapies [74]. Common side effects include flu-like symptoms, headache, back pain, and fever; later effects may involve elevated liver enzymes. No serious adverse events were reported. No clinical studies have been conducted on Eritoran, Resatorvid, or GSK1795091 in the context of PD or COPD. TLR4-targeting therapies may offer a more selective and effective way to control inflammation in PD, particularly by modulating the immune response triggered by *P. gingivalis* and other pathogens. However, the pertussis vaccine, while TLR4-related, is not considered relevant for PD or COPD treatment, as its primary function is to induce immunity against Bordetella pertussis, not to modulate chronic inflammation in these diseases [75].

Furthermore, three drug candidates were identified in association with caspase-8: CHEMBL375563, Nivocasan, and Conatumumab. (20S)-Protopanaxadiol, registered as CHEMBL375563, is a bioactive triterpenoid metabolite derived from ginsenosides. Nivocasan irreversibly arrests caspase-1 activity and inhibits that of caspase-8, effectively blocking apoptotic pathways [76]. Conatumumab, an investigational fully human IgG1 monoclonal antibody, targets death receptor 5 (DR5) and induces apoptosis via caspase activation [77]. However, to date, no clinical or preclinical studies have explored the relevance of these compounds in PD or COPD.

Since our study is entirely based on computational analyses, the findings serve as a foundation for future experimental research. Although none of the 12 potential drug candidates (except pertussis vaccine) identified have been studied in clinical trials for either PD or COPD, they highlight key molecular targets shared by both conditions. In particular, targeting inflammatory pathways through agents modulating PERK, IL-1β, TLR4, and caspase activity appears to be a promising therapeutic strategy. These results open new avenues for both in vitro and in vivo studies, which are essential to validate the bioinformatic predictions and to assess the efficacy and safety of these candidates in relevant disease models.

Machine learning has advanced diagnosis and prognosis in both COPD and periodontitis by improving biomarker identification and disease prediction. While most studies focus on each disease separately [78,79,80,81,82,83], our work integrates PANoptosis-related genes across both, providing novel insights into shared mechanisms and therapeutic targets. We applied machine learning (XGBoost) to classify disease and control samples based on PANoptosis-related core genes, demonstrating their potential utility in improving biomarker-based diagnosis and prognosis of both COPD and periodontitis. This approach highlights the translational relevance of our findings and supports future interdisciplinary research integrating computational methods with molecular biology.

Our study has several limitations that should be explicitly acknowledged. First, as a bioinformatics-based analysis, it lacks experimental validation. The findings have not yet been confirmed at the transcriptional or protein level using laboratory methods such as qPCR, Western blotting, or immunohistochemistry. Therefore, further in vitro and in vivo studies are necessary to validate the proposed biomarkers and mechanistic pathways. Second, the sample size of the COPD dataset was smaller than that of the PD dataset, which may reduce statistical power and the robustness of cross-disease comparisons. Third, the analysis was limited by the scope and quality of publicly available transcriptomic data, which may affect the generalizability of the findings to broader populations. Despite these limitations, our study contributes to translational research by identifying core seven PANoptosis-related genes shared between PD and COPD, highlighting their potential as novel biomarkers and therapeutic targets. Unlike conventional clinical parameters that indicate disease only after onset, these core genes may serve as early molecular indicators of disease activity, paving the way for more accurate, non-invasive, and predictive diagnostic tools. Furthermore, our drug–gene interaction analysis offers a foundation for targeted therapeutic development. Future studies should focus on validating these core PANoptosis-related genes in clinical samples such as saliva and gingival crevicular fluid, and on exploring their diagnostic, prognostic, and therapeutic utility through functional and longitudinal studies.

In conclusion, our study reveals a complex interplay between *MEFV*, *NLRC4*, *CASP8*, *IL1B*, *ACO1*, *CYCS*, and *HSPA4* in the pathogenesis of PD and COPD. These seven genes are involved in key inflammatory pathways, such as inflammasome activation, iron metabolism, oxidative stress, and cell death. The shared molecular mechanisms underlying both diseases suggest that targeting these pathways could provide novel therapeutic strategies for managing PD and COPD. These findings warrant further exploration of the implicated genes, with the potential to inform novel treatment strategies for both conditions.

## 5. Conclusions

This study provides the first bioinformatic evidence linking PANoptosis to both PD and COPD through seven core genes. These findings offer a promising basis for the development of diagnostic biomarkers and targeted therapies aimed at modulating chronic inflammation in both conditions. While experimental validation is still required, the identification of shared molecular pathways opens new directions for translational research, particularly in the context of host-modulation strategies. Future efforts should prioritize validating these targets in clinical samples and assessing their relevance for personalized medicine.

## Figures and Tables

**Table 1:** Detailed summary of GEO gene expression datasets used in this study, including accession number, tissue type, platform, organism, case versus control distribution, and applied inclusion/exclusion criteria.

GEO Accession	Tissue Type	Platform	Organism	Case (Disease)	Control (Healthy)	Inclusion/Exclusion Criteria
GSE16134	Gingival tissue	GPL570	Homo sapiens	241 PD	69	Included: diagnosed PD vs. healthy gingival tissue; Excluded: non-human, unclear diagnosis
GSE10334	Gingival tissue	GPL570	Homo sapiens	183 PD	64	Included: confirmed PD; Excluded: datasets with <10 subjects
GSE27597	Lung tissue	GPL5175	Homo sapiens	48 COPD	16	Included: COPD patients vs. matched controls; Excluded: non-human
GSE38974	Lung tissue	GPL4133	Homo sapiens	23 COPD	9	Included: COPD diagnosis; Excluded: insufficient annotation
GSE76925	Lung tissue	GPL10558	Homo sapiens	111 COPD	40	Included: human lung samples; Excluded: non-COPD respiratory diseases
GSE106986	Lung tissue	GPL13497	Homo sapiens	14 COPD	5	Included: COPD vs. healthy; Excluded: samples with missing phenotype

## Data Availability

The original contributions presented in this study are included in the article/Supplementary Materials. Further inquiries can be directed to the corresponding author.

## Supplementary Materials

The following supporting information can be downloaded at: https://www.mdpi.com/article/10.3390/genes16091027/s1, Table S1: DEGs results for PD. Table S2: DEGs results for COPD. Table S3: Panoptosis gene list.
